# Tobacco cessation in patients with multiple chronic conditions: nutritional strategies as an additional tool in treatment

**DOI:** 10.47626/2237-6089-2021-0427

**Published:** 2024-01-08

**Authors:** Marcela Melquíades de Melo, Arthur da Silva Gomes, Thayzis de Paula Silva, Arise Garcia de Siqueira Galil, André Netto Bastos, Aline Silva de Aguiar

**Affiliations:** 1 Programa de Pós-Graduação em Saúde Coletiva Faculdade de Medicina Universidade Federal de Juiz de Fora Juiz de Fora MG Brazil Programa de Pós-Graduação em Saúde Coletiva , Faculdade de Medicina , Universidade Federal de Juiz de Fora , Juiz de Fora , MG , Brazil .; 2 Universidade Federal Fluminense Niterói RJ Brazil Universidade Federal Fluminense , Niterói , RJ , Brazil .; 3 Universidade Federal de Ouro Preto Ouro Preto MG Brazil Universidade Federal de Ouro Preto , Ouro Preto , MG , Brazil .

**Keywords:** Chronic diseases, smoking, smoking cessation, nutrition, food and nutrition education

## Abstract

**Objective:**

To evaluate the impacts of a nutritional education intervention for patients with multiple chronic conditions during smoking cessation.

**Methods:**

The non-probabilistic sample comprised 18 adults and seniors of both sexes recruited from a smoking cessation treatment group. At the beginning of treatment, smoking history, degree of dependence, and stage of motivation were assessed. Degree of craving was evaluated weekly for the 1st month. Anthropometric and biochemical assessments were conducted at baseline, at 1 month, and at 3 months. Dietary intake was assessed with the “How is your diet?” questionnaire. The nutritional intervention was delivered in three sessions. The themes covered were energy balance and physical activity, healthy eating, and the importance of fruit and vegetables in the diet. Statistical analysis was conducted with the Shapiro Wilk test of normality, the paired *t* test, and the Wilcoxon or Mann-Whitney *U* tests (significance ≤ 0.05).

**Results:**

Most people (55.6%) in the intervention group had a high degree of smoking dependence, while the frequency in the control group was 22.2%. Degree of craving decreased significantly after 1 month of treatment (p = 0.017). After 3 months, both groups had a positive variation in mean body weight, although below 3%. In both groups, the average percentage of weight gain was less than 3%, suggesting that delivery of the nutritional education sessions and the nutritionist’s use of the protocol proposed by the Instituto Nacional de Câncer (INCA) helped to control weight gain. Blood glucose and homeostasis model assessment-insulin resistance (HOMA-IR) both increased significantly in the intervention group (p = 0.15 and p = 0.50, respectively).

**Conclusion:**

Greater proximity and more frequent intervention by a nutritionist assists and encourages healthy eating practices during the smoking cessation process, which can benefit individuals’ control of chronic diseases over the long term.

## Introduction

In the year 2016, tobacco consumption was responsible for the deaths of more than 7.1 million people worldwide, 5.1 million men and 2 million women. In Brazil, 13% of all deaths in the country can be attributed to cigarettes and smoking is responsible for 443 deaths per day. ^1^ In 2030, it is estimated that more than eight million deaths annually will be due to smoking worldwide, constituting an important public health problem. ^2^

Smoking is recognized as a chronic disease caused by dependence on the nicotine present in tobacco products. In addition, it is also considered the largest single preventable cause of illness and early death worldwide, comprising an important risk factor for development of other chronic diseases. ^3^

Continuous exposure to approximately 4,720 toxic substances makes smoking the cause of cardiovascular diseases, cancer, and chronic obstructive respiratory diseases. ^4^ Chronic non-communicable diseases (NCDs) are responsible for the greatest burden of diseases. The number of people living with two or more of these chronic conditions, known as multiple chronic conditions (MCC), is increasing. ^5^ Promoting smoking cessation is an important strategy for reduction of morbidity and mortality associated with smoking-related diseases and adequate control of chronic NCDs. ^6^ Nicotine is a psychoactive substance found in tobacco that stimulates the central nervous system, generating sensations related to motivation, attention, pleasure, and reward. ^7^ Therefore, most smokers attempting to quit pass through repeated cycles of abstinence followed by relapse to smoking before they achieve long-term abstinence. ^8^ In addition, the quitting process is complex since it causes a withdrawal syndrome. The symptoms presented are depressed mood, insomnia, irritability, anxiety, difficulty concentrating, restlessness or impatience, bradycardia, increased appetite, and weight gain. ^9^

Weight gain constitutes a relevant risk factor for relapse. Encouraging healthy eating practices with a nutritional approach during smoking cessation promotes better food choices and helps to reduce anxiety about weight gain resulting from tobacco withdrawal, encouraging cessation. Regardless of the effect on body weight and other anthropometric variables, continued contact with advisors can reduce participants’ anxiety about weight gain and thus encourage continued cessation despite weight gain. ^10^ Individuals should be encouraged to allay their concerns about weight gain in order to obtain other health gains by quitting smoking and not only attributing value to possible body weight gain. ^11^

Nutritional intervention actions have promoted improvements in eating habits, with greater consumption of fruits and vegetables and cereals for breakfast. ^10^ Moreover, foods are used to reduce the urge to smoke and remain abstinent. Among individuals being monitored for smoking cessation, the most often cited foods used as a resource to control the urge to smoke were fruits, coffee, and candies. It was also observed that abstinent individuals increased consumption of water to help control their craving, which is an aspect that should be encouraged, since it can contribute to people’s weight control. ^12^ Many smokers do not stop cigarette smoking until they have already developed smoking-related complications, ^8^ such as chronic conditions, especially those with longer duration of smoking, demanding urgent smoking cessation. ^13^ From such a perspective, smoking cessation combined with an individualized diet may be a viable approach to weight management and maintenance of abstinence, ^14^ which are extremely relevant in patients with MCC.

Given this, initiatives for nutritional education during smoking cessation could assist in food choices and control of weight gain. ^10 , 12^ The present study aims to assess the impacts of a nutritional education intervention on the smoking cessation process in patients with MCC at the Unidade de Atendimento Integral ao Tabagismo (UAIT), run by the Fundação Instituto Mineiro de Estudos e Pesquisas em Nefrologia (IMEPEN), at the Universidade Federal de Juiz de Fora (UFJF), Minas Gerais, Brazil.

## Methods

This is a clinical intervention study, conducted with a non-probabilistic sample, registered and published on the Brazilian Clinical Trials Registry (ReBEC) platform, under record number RBR-83jr3x. This study is part of a larger project with funding from the Fundação de Amparo à Pesquisa do Estado de Minas Gerais (FAPEMIG) to conduct a range of different nutritional interventions among smoking cessation group participants with MCC. This article presents the results achieved by participants who were enrolled on an educational nutritional intervention.

### Selection and description of participants

The non-probabilistic sample consisted of adult and senior smokers of both sexes recruited from a smoking cessation treatment group at the IMEPEN Foundation UAIT, which exclusively serves patients with at least one chronic condition in addition to smoking. The study included adult and senior (18 to 75 years) individuals of both sexes with at least two chronic pathologies in addition to smoking (diabetes, high blood pressure, cardiovascular disease, and chronic obstructive pulmonary disease), characterizing MCC, participating in a smoking cessation treatment group at UAIT. Exclusion criteria for this study were individuals with cancer, infectious diseases, liver diseases, and chronic kidney disease (CKD) undergoing conservative or dialysis treatment and those who already had an individualized nutritional prescription. Individuals who did not wish to participate in the study were not included.

The UAIT smoking treatment groups follow the Instituto Nacional de Câncer (INCA) protocol with multidisciplinary follow-up. The initial month of follow-up consists of four weekly meetings, followed by fortnightly meetings in the 2nd month, and then monthly meetings until the end of treatment. In addition to these sessions, the team holds an awareness session with the participants 1 week before starting the INCA protocol. The third weekly session comprises the nutrition and physical activity module proposed by INCA, during which the team makes patients aware of the impacts of smoking cessation, nutritional strategies to assist in smoking cessation, and the importance of physical activity. ^15^

According to the INCA protocol, ^15^ patients can be medicated for smoking cessation according to the medical recommendation with nicotine replacement therapy (transdermal patch) and/or bupropion. In addition to these medications, each patient continues to use any medications previously prescribed for preexisting chronic conditions.

### Technical information

The educational nutritional intervention, on which this article focuses, consisted of three sessions held during the fourth weekly meeting, the second fortnightly meeting, and the first monthly meeting. The meetings took place collectively and lasted approximately 20 minutes. Participants were present at a minimum of two of the meetings, with only one absence being accepted. The methodology and themes covered at each meeting were pre-defined. The theme of the first meeting was “energy balance and physical activity,” aiming to explain the effects of smoking on energy balance. The second meeting dealt with “healthy eating,” to provide guidance on the composition of a healthy balanced diet. The third meeting focused on “the importance of fruit and vegetables in the diet,” encouraging consumption of at least five portions per day, and on “the contribution of fats to weight gain,” encouraging reduction of fat intake. During these meetings, the researchers used resources such as illustrative pamphlets and conversation circles. Sessions began with questions about the previous topics for clarification of possible doubts.

Patients responded to an initial medical history form to collect their smoking history. The model for assessing the stages of change developed by Prochaska and DiClemente ^16^ was used to assess motivation at the start of treatment. The Fagerstrom Test for Nicotine Dependence (FTND) ^17^ was used to measure nicotine dependence. Degree of craving was assessed at the beginning of treatment and after 1 month with the Questionnaire on Smoking Urges – Brief Brazilian version (QSU) and classified as minimal (0 to 13 points), mild (14 to 26 points), moderate (27 to 42 points), or intense (43 or more points). ^18^ The questionnaire was used during the intensive phase, which is the 1st month of the approach proposed by INCA, the period of greatest craving.

The initial nutritional status evaluation consisted of an anthropometric assessment with measurement of body weight, height, and waist circumference (WC) and was repeated after 1 and 3 months. Bodyweight was determined using a digital scale (Tanita ^®^ ) with the subject assessed barefoot and wearing light clothing. Height was measured with a portable stadiometer (Alturexata ^®^ ) with the patient barefoot, standing upright, with head erect and heels together, and in contact with the vertical rod of the stadiometer. To assess nutritional status, the body mass index (BMI) was calculated as weight (kg)/height (m ^2^ ) and classified accordingly to the World Health Organization (WHO) ^19^ criteria for adults or the elderly. ^20^ Participants’ WC was measured with a flexible measuring tape at the midpoint between the last rib and the iliac crest. The results were interpreted using the cutoff points proposed by the International Diabetes Federation, ^21^ where WC ≥ 94 cm and ≥ 80 cm indicates a risk of cardiovascular disease for men and women, respectively. ^21 , 22^

Indicators suggested by ordinance no. 571, of April 5, 2013, on assessment and monitoring of care for smokers were used to measure the success of the smoking cessation treatment and drop-out rates. ^23^

The “How is Your Diet?” questionnaire (Como Está sua Alimentação? [CESA], from Portuguese), ^24^ produced by the Health Ministry for assessment of the adequacy of food consumption, was administered to gather information on consumption of fruits, fats, processed products, fried foods, and water. This questionnaire was administered at the beginning and at the end (after 3 months) of the intervention, making it possible to compare the change in consumption (decreased, increased, or unchanged) of the food items analyzed in the questionnaire using absolute and relative frequencies. Only the 3-month assessment period was analyzed because assessment after just 1 month of treatment was considered too short a period for dietary changes to manifest. The UAIT’s doctor or nutritionists requested the following laboratory tests at the beginning of the follow-up and after 1 and 3 months of treatment: blood count, lipid profile (total cholesterol and fractions and triglycerides), blood glucose, uric acid, creatinine, cortisol, insulin, and C-reactive protein (CRP). Blood samples were drawn after 8 to 12 hours of fasting, between 7 a.m. and 8 a.m., at the Laboratório Cortes Villela, São Pedro Unit. The same laboratory performed the analyses. The control group comprised participants from UAIT smoking treatment groups who did not receive the nutritional intervention.

The present study was conducted with humans in accordance with the World Medical Association’s code of ethics for experiments involving humans. Thus, we declare that all individuals who participated in the research granted their consent and that their privacy rights were observed. This research was approved by the UFJF Research Ethics Committee, under ethical appraisal submission certificate number 55440716.9.0000.5147, opinion 1.693.278.

### Statistics

SPSS 23.0 software was used to perform statistical analysis. The descriptive analyses used were mean, standard deviation (SD), median, and confidence interval. The Shapiro Wilk test was used to verify the normality of the data. The Mann-Whitney *U* test was used to compare variables between the control group and the intervention group. Wilcoxon or paired *t* tests were used to compare baseline values with data collected after 1 month and 3 months. The level of significance adopted in all analyses was 0.05. Student’s *t* test was used to compare mean sum of weekly QSU results after the 1st month of follow-up.

## Results

Data were collected from smoking cessation courses delivered between April 2017 and May 2018. This article presents data related to smokers who participated in groups that received nutritional education interventions. The control group and the intervention group were composed of nine participants each. Table 1 shows the characteristics of smokers by intervention or control group.

**Table 1:** Characteristics of the participants in the intervention and control groups at the beginning of follow-up

	Intervention group	Control group	Total	p-value
Sex, n (%)				
Female	7 (77.8)	5 (55.6)	12 (66.7)	0.317*
Male	2 (22.2)	4 (44.4)	6 (33.3)	
Anthropometric data, mean (SD)				
Body weight (kg)	66.39 (13.06)	71.29 (13.59)	68.84 (13.17)	0.447 ^†^
Body mass index (kg/m ^2^ )	26.35 (3.58)	27.06 (3.68)	26.71 (3.54)	0.686 ^†^
Waist circumference (cm)	93.11 (13.97)	93.00 (11.44)	93.06 (12.44)	0.986 ^†^
Females	90.43 (13.87)	95.20 (14.55)	92.42 (13.71)	0.577 ^†^
Males	102.5 (13.43)	89.3 (1.53)	94.6 (9.91)	0.396 ^†^
Smoking history				
Number of cigarettes per day, n (%)				0.257*
< 20	6 (66.7)	8 (88.9)	14 (77.8)	
≥ 20	3 (33.3)	1 (11.1)	4 (22.2)	
Degree of smoking dependence, n (%)				0.425*
Very low and low	3 (33.3)	4 (44.4)	7 (38.9)	
Moderate	1 (11.1)	3 (33.3)	4 (22.2)	
High and very high	5 (55.6)	2 (22.2)	7 (38.9)	
Addiction duration (years), mean (SD)	46.8 (4.5)	36.2 (9.5)	41.5 (9.0)	0.008 ^†^
Previous attempts to quit, mean (SD)	2.2 (1.3)	1.8 (1.3)	2.1 (1.3)	0.564 ^†^
Motivation stage, n (%)				0.565*
Contemplation	0 (0.0)	1 (11.1)	1 (5.5)	
Preparation	5 (55.6)	5 (55.6)	10 (55.6)	
Action	4 (44.4)	3 (33.3)	7 (38.9)	
Drug therapy, n (%)				0.072*
Nicotine replacement therapy	1 (11.1)	3 (33.3)	4 (22.2)	
Bupropion	3 (33.3)	2 (22.2)	5 (27.8)	
Nicotine replacement therapy + Bupropion	4 (44.4)	0 (0.0)	4 (22.2)	
No prescription medication	1 (11.1)	4 (44.4)	5 (27.8)	
Cessation, week 4, n (%)	4 (44.4)	2 (22.2)	6 (33.3)	0.256*
Cessation, week 12, n (%)	5 (55.6)	2 (22.2)	7 (38.9)	0.073*
Chronic diseases, n (%)				
Systemic arterial hypertension	8 (88.9)	7 (77.8)	15 (83.3)	0.527*
Acute myocardial infarction	3 (33.3)	2 (22.2)	5 (27.8)	0.599*
Stroke	1 (11.1)	2 (22.2)	3 (16.7)	0.527*
Diabetes mellitus	2 (22.2)	7 (77.8)	9 (50.0)	0.018*
Chronic obstructive pulmonary disease	2 (22.2)	0 (0.0)	2 (11.1)	0.220*

SD = standard deviation.

* Chi-square test; ^†^ Student’s *t* test.

Patients (n = 18) were, on average, 62.4±5.4 years old, ranging from 54 to 72 years. Most of them were female (n = 12; 66.7%), married or living in a stable relationship (n = 10; 55.6%), and studied for at least 8 years (n = 10; 55.6%). The most prevalent degree of dependence was high to very high (n = 7; 38.9%). Arterial hypertension was the most prevalent chronic condition (n = 15; 83.3%), followed by diabetes mellitus (n = 9; 50%).

The proportion of treatment success, i.e., the proportion of smokers who had stopped smoking by the fourth session, was 33.3%. At the 12th week of treatment, the cessation rate was 38.9%. Table 1 shows the characteristics of smokers by intervention or control group.

The craving assessment at 1 month of follow-up, for all participants regardless of group, showed that the degree of craving had significantly decreased (p = 0.017) with an initial average of 27.7 points and a final average of 16.3 points. When the groups were analyzed separately, the craving reduction remained. In the intervention group, the initial average was 26.3 points and the final average was 15.6 points. In the control group, the average initial craving value was 29.1 and the final value was 17.0 points. However, there were no statistical differences in the analysis by group. The average QSU scores showed that the degree of craving decreased from moderate to mild craving in both groups. Table 2 shows the QSU results over the 1st month of follow-up.

**Table 2:** Weekly mean QSU and SD over the 1st month of follow-up

QSU	Intervention mean (SD)	Control mean (SD)	Total mean (SD)
Week 1	26.3 (11.2)	29.1 (18.9)	27.7 (15.1)
Week 2	17.9 (6.9)	30.7 (16.7)	24.3 (14.0)
Week 3	15.7 (4.3)	26.0 (20.5)	20.8 (15.2)
Week 4	15.6 (6.9)	17.0 (6.4)	16.3 (6.5)*

SD = standard deviation; QSU = Questionnaire on Smoking Urges.

* Student’s *t* test (p = 0.017).

The initial nutritional status assessment by BMI according to age group showed that the majority of the control group were overweight/obese (n = 7; 77.8%). In the intervention group, it was observed that the majority (n = 5; 55.6%) had healthy weight and 33.3% were overweight/obese. Despite the change in average body weight, there was no change in BMI nutritional status category in either group.

All participants were classified as having high cardiovascular risk according to their WC. In the intervention group, 77.8% of participants were classified as very high risk.

Analysis of the change in body weight revealed positive weight change after 3 months, constituting weight gain regardless of group. The percentage change in average body weight was below 3% in both groups and Student’s t test revealed no significant difference, as shown in Table 3 . Descriptive data on changes in food consumption are presented in Table 4 .

**Table 3:** Mean and SD of change in body weight in the follow-up period

	Intervention group	Control group	Total
Change in body weight after 1 month (kg)	-0.22 (1.13)	0.26 (1.28)	0.006 (1.19)
Change in body weight after 3 months (kg)	1.47 (2.23)	1.75 (2.87)	1.57 (2.33)
Percentage of body weight change after 3 months (%)	2.27 (3.36)	2.49 (4.08)	2.35 (3.40)

SD = standard deviation.

**Table 4:** Changes in food consumption comparing the beginning and end of the follow-up period in the intervention and control groups

Food consumption/group	Decreased n (%)	Increased n (%)	Unchanged n (%)
Fruit consumption			
Intervention	1 (12.5)	5 (62.5)	2 (25.0)
Control	3 (50.0)	0 (0.0)	3 (50.0)
Fat consumption			
Intervention	2 (25.0)	1 (12.5)	5 (62.5)
Control	1 (16.7)	2 (33.3)	3 (50.0)
Consumption of fried foods and processed foods			
Intervention	3 (37.5)	4 (50.0)	1 (12.5)
Control	1(16.7)	3 (50.0)	2 (33.3)
Water consumption			
Intervention	0 (0.0)	3 (37.5)	5 (62.5)
Control	1 (16.7)	2 (33.3)	3 (50.0)

In the intervention group, comparison of initial biochemical parameter values with values after 1 month revealed increases in blood glucose, homeostasis model assessment-insulin resistance (HOMA-IR), cortisol, HDL, creatinine, and CRP. The values of insulin, HOMA-Beta, total cholesterol, LDL, VLDL, triglycerides, and uric acid decreased. However, when the initial values were compared to values at 3 months, there were increases in insulin and total cholesterol.

When comparing the initial values with values after 1 month for the control group, there were increases in blood glucose, cortisol, VLDL, triglycerides, uric acid, creatinine, and CRP. Values for insulin, HOMA-Beta, total cholesterol, HDL, and LDL, reduced. In contrast, when the values at 1 month and 3 months were compared, insulin and total cholesterol increased and cortisol and CRP decreased.

Cortisol values increased when comparing baseline (10.50 and 8.41 mcg/dL, intervention and control respectively) with values after 1 month (11.29 and 10.15 mcg/dL), during the intensive phase. Comparing the values from 1 month with values at 3 months, there was a reduction in cortisol (11.12 and 7.93 mcg/dL).

Glycemia and cortisol both increased when comparing baseline (104.0 and 89.0 mg/dL intervention and control, respectively) to 1 month (113.0 and 100.0 mg/dL) and reduced when comparing the 1st month to the 3rd month (112.0 and 97.5 mg/dL). However, the final blood glucose value was higher than the initial value in both groups.

Significant differences between groups were observed only in initial and 1-month total cholesterol (p = 0.01 and 0.04, respectively). In the intervention group, blood glucose and the HOMA-IR index increased significantly after 3 months (p = 0.01 and 0.05, respectively). In the control group, biochemical parameters did not exhibit statistically significant differences during follow-up. Table 5 shows the results of the biochemical tests.

**Table 5:** Biochemical parameters at baseline and after 1 month and 3 months in the intervention and control groups

Parameter	Intervention group		Control group		Reference value
	Median	95%CI	Median	95%CI	
Initial blood glucose	104.00	93.87-126.35	89.00	59.51-140.48	60 to 99 mg/dL
Blood glucose, 1 month	113.00	92.13-148.31	100.00	65.10-165.89	
Blood glucose, 3 months*	112.00	91.49-167.39	97.50	75.37-127.63	
Initial insulin	8.20	5.62-13.26	8.70	0.64-18.51	2.6 to 24.9 mcUI/mL
Insulin, 1 month	7.80	5.66-12.62	7.45	-20.58-57.53	
Insulin, 3 months	8.90	5.72-20.95	13.25	0.98-21.66	
Initial HOMA-IR	2.00	1.37-3.85	1.85	-0.99-6.19	BMI up to 25 kg/m ^2^ : 0.4 to 2.9; BMI between 25 and 29.9kg/m ^2^ : 0.4 to 4.3; BMI over 30 kg/m ^2^ : 0.7 to 8.2
HOMA-IR, 1 month	2.20	1.37-4.32	1.85	-9.76-23.16	
HOMA-IR, 3 months*	2.30	1.28-8.18	3.15	0.03-5.77	
Initial HOMA-Beta	80.00	47.92-111.74	104.55	34.63-167.07	100%
HOMA-Beta, 1 month	54.10	37.27-104.66	73.65	-16.97-208.67	
HOMA-Beta, 3 months	70.40	36.23-130.65	95.35	-31.31-265.16	
Initial cortisol	10.50	7.57-11.92	8.41	3.84-15.39	3.70 to 19.40 mcg/dL
Cortisol, 1 month	11.29	9.09-13.46	10.15	8.08-11.65	
Cortisol, 3 months	11.12	9.39-14.89	7.93	2.71-14.51	
Initial total cholesterol ^†^	227.00	221.60-245.94	210.00	163.50-252.49	Up to 190 mg/dL
Total cholesterol, 1 month ^†^	217.00	189.95-259.82	204.00	141.06-265.94	
Total cholesterol, 3 months	244.00	206.56-256.10	205.50	163.58-235.42	
Initial HDL-c	48.00	42.46-66.65	45.00	27.30-64.69	41 to 59 mg/dL
HDL-c, 1 month	51.00	41.71-67.18	41.50	21.25-67.25	
HDL-c, 3 months	52.00	47.03-69.19	41.50	29.59-58.91	
Initial LDL-c	145.60	88.48-164.86	140.50	102.09-175.31	100 to 129 mg/dL
LDL-c, 1 month	126.00	99.39-176.87	131.30	86.50-179.39	
LDL-c, 3 months	131.60	111.48-153.72	124.20	80.77-160.12	
Initial triglycerides	157.00	112.49-215.28	123.50	73.99-159.01	Less than 150 mg/dL
Triglycerides, 1 month	142.00	96.24-226.87	136.00	65.69-197.31	
Triglycerides, 3 months	157.00	113.52-292.69	179.00	79.92-268.08	
Initial uric acid	5.77	4.05-7.27	5.05	3.81-6.02	Females: 2.6 to 6.0 mg/dL; males: 3.5 to 7.2 mg/dL
Uric acid, 1 month	5.63	3.92-7.61	5.55	3.47-6.86	
Uric acid, 3 months	5.12	4.17-6.95	5.72	3.78-6.87	
Initial creatinine	0.92	0.77-1.11	0.82	0.29-1.19	Females: 0.6 to 1.1 mg/dL; males: 0.9 to 1.3 mg/dL
Creatinine, 1 month	0.93	0.82-1.21	0.94	0.63-1.20	
Creatinine, 3 months	0.92	0.79-1.16	0.95	0.74-1.15	
Initial PCR	0.42	0.27-1.19	0.39	-0.09-1.06	Up to 0.60 mg/dL
PCR, 1 month	0.45	0.13-2.68	0.51	-1.32-3.75	
PCR, 3 months	0.67	0.25-1.95	0.37	-0.01-0.80	

95%CI = 95% confidence interval; BMI = body mass index; HOMA-IR = homeostasis model assessment-insulin resistance; PCR = polymerase chain reaction.

* Significant difference with Wilcoxon test; ^†^ Significant difference with Mann-Whitney *U* test;

## Discussion

In the present study, most participants were female and classified as in the preparation stage of the motivation scale at the beginning of the follow-up. The majority of participants in the intervention group had a high or very high degree of smoking dependence. The smokers in the control group had a higher percentage of overweight/obesity at the beginning of treatment and had a higher average body weight than smokers in the intervention group. However, they showed a higher frequency of increased consumption of fats and a lower frequency of increased consumption of fruits. Among biochemical parameters, blood glucose increased significantly in the intervention group. A more significant increase in cortisol was observed in the intensive phase, the 1st month of the approach proposed by INCA. ^15^

Although smoking is more frequent among men, ^15^ most participants were female. This can be explained by women seeking health services and health care more frequently. Among the female smoking population, weight gain related to smoking cessation is the main reason why they are unable to stop smoking or prone to relapse. ^25^

It is also noteworthy that many smokers are already overweight when beginning treatment for smoking cessation. ^26^ In this study, it was possible to observe this situation, since more than half of the smokers who started the treatment were already overweight/obese, especially in the control group.

In a study in the United States, being a heavy smoker (more than 25 cigarettes/day) was a risk factor for weight gain. Among the smokers studied, only 22.2% had consumption exceeding 20 cigarettes/day. Smokers who smoke more cigarettes per day weigh more and are more likely to be obese than those who smoke fewer cigarettes per day. It is suggested that greater consumption of cigarettes may reflect a behavioral disorder related to appetite, generating excessive consumption of both nicotine and food, making obesity more likely. ^27^

A study by Tuovinen et al. ^28^ showed that the greatest obstacle to abstinence for smokers with a high degree of nicotine dependence is the dependence itself, more than the influence of weight issues. The intervention group had a higher percentage of high to very high dependence, permanence, and abstinence at 3 months. However, this did not represent treatment losses or abandonment, or different interventions. The nutritional program during cessation helps to reduce concerns about body weight and the perception of increased body weight, encouraging participants to remain abstinent regardless of body weight gain. ^11^ The concern about weight gain after cessation constitutes a stronger predictor of relapse than body weight gain itself. ^29^

Higher initial glycemia was observed among smokers in the intervention group, but without statistical difference for the control group. It should be noted that there was a higher percentage of patients with diabetes in the control group, with a significant difference compared with the intervention group (p = 0.018). Patients with a previous diagnosis of diabetes mellitus were already taking medication prescribed to control diabetes when they started treatment for smoking.

There was a statistical difference between initial glycemia and glycemia after 3 months in the intervention group. The HOMA-IR index, which reflects insulin resistance, also increased. Nicotine can influence the function of β cells directly or indirectly, through parasympathetic ganglia, favoring insulin resistance. High concentrations of nicotine inhibit glucose-induced insulin secretion and insulin sensitivity has been shown to improve with smoking cessation, occurring in conjunction with normalization of phosphorylation of insulin receptor substrate 1. ^29^ Also, the lowest glycemia level was observed in the control group, accompanied by higher insulin and HOMA-IR after 3 months.

Serum cortisol showed a more significant increase in the 1st month of follow-up and after 3 months had reduced in both groups. Anxiety-provoking situations such as threats, lack of control, and anticipation of aversive events are associated with increased cortisol release. Behavioral tasks involving novelty, uncertainty, and negative affect are also related to increases in cortisol. ^30^ During the intensive phase, comprising the 1st month of smokers’ monitoring for cessation, such aspects are probably present and accentuated, explaining the initial elevation of cortisol observed.

The reduction in cortisol observed after 3 months may be related to better control of these sensations. Added to this is the effect of nicotine, which stimulates cholinergic receptors in the hypothalamus, stimulating corticotropic releasing factors, which initiate the hypothalamic-pituitary-adrenocortical cascade that leads to production of cortisol. ^31^

## Conclusion

The average percentage weight gain was less than 3%, confirming that delivery of the nutritional education sessions and the nutritionist’s use of the protocol proposed by the INCA helped in the control of weight gain. Therefore, greater proximity and more frequent intervention by a nutritionist assists and encourages healthy eating practices during the smoking cessation process, which can benefit individuals’ control of chronic diseases over the long term.

Limitations of this study include non-randomization, the sample size, the non-probabilistic design, and the limited period of follow-up for verifying changes in smokers’ eating habits and maintenance of abstinence over the long term. In an attempt to minimize these constraints, the time and period of data collection are highlighted. It is essential to highlight that members the population studied have difficulties with adherence to the treatment, since they are already maintaining their smoking habit despite cardiovascular and metabolic complications. This therefore justifies the importance of this study, since it seeks strategies that help these smokers to achieve cessation.
